# Disruption in the Blood-Brain Barrier: The Missing Link between Brain and Body Inflammation in Bipolar Disorder?

**DOI:** 10.1155/2015/708306

**Published:** 2015-05-13

**Authors:** Jay P. Patel, Benicio N. Frey

**Affiliations:** ^1^MiNDS Neuroscience Graduate Program, McMaster University, Hamilton, ON, Canada L8S 4L8; ^2^Mood Disorders Program and Women's Health Concerns Clinic, St. Joseph's Healthcare, Hamilton, ON, Canada L8P 3B6; ^3^Department of Psychiatry and Behavioural Neurosciences, McMaster University, Hamilton, ON, Canada L8S 4L8

## Abstract

The blood-brain barrier (BBB) regulates the transport of micro- and macromolecules between the peripheral blood and the central nervous system (CNS) in order to maintain optimal levels of essential nutrients and neurotransmitters in the brain. In addition, the BBB plays a critical role protecting the CNS against neurotoxins. There has been growing evidence that BBB disruption is associated with brain inflammatory conditions such as Alzheimer's disease and multiple sclerosis. Considering the increasing role of inflammation and oxidative stress in the pathophysiology of bipolar disorder (BD), here we propose a novel model wherein transient or persistent disruption of BBB integrity is associated with decreased CNS protection and increased permeability of proinflammatory (e.g., cytokines, reactive oxygen species) substances from the peripheral blood into the brain. These events would trigger the activation of microglial cells and promote localized damage to oligodendrocytes and the myelin sheath, ultimately compromising myelination and the integrity of neural circuits. The potential implications for research in this area and directions for future studies are discussed.

## 1. Physiology of the Blood-Brain Barrier

### 1.1. Structure of the BBB

The characterization of blood-brain barrier (BBB) began in 1885 with Paul Ehrlich's reports that various water-soluble dyes failed to stain the brain and spinal cord upon injection into the circulatory system, which he attributed to the lower affinity for the dye by the CNS [1, 2]. Later in 1898, Biedl and Kraus demonstrated that only injection of bile acids directly into the brain caused symptoms including seizures and coma, but not when injected into the circulatory system [2]. In 1900, Lewandowsky demonstrated a similar effect using potassium ferrocyanide and attempted to describe this with the term* bluthirnschranke *(blood-brain barrier) [1]. Further experimentation by Goldmann, a student of Ehrlich, demonstrated that trypan blue when injected into the cerebrospinal fluid (CSF) stained CNS tissue, contradicting Ehrlich's dye affinity hypothesis and lending support to the notion that there is a barrier between the circulatory system and the CNS [2]. Then in 1967, with newly available electron microscopy technology, Reese and Karnovsky demonstrated at the ultrastructural level that horseradish peroxidase (HRP) was unable to enter the CNS due to the presence of tight junctions (TJ) [3]. This showed the continuous nature of the BBB in the CNS and led Reese and Karnovsky to conclude that the BBB existed at the level of the endothelial cells.

Acting as a diffusion barrier, the BBB is composed primarily of brain endothelial cells, astrocyte end-feet, pericytes, perivascular macrophages, and a basal membrane. Its barrier is a result of a tightly sealed monolayer of endothelial cells with TJ and adherens junctions (AJ) forming the seal between cells at junctional complexes. The basal membrane and astrocyte end-feet contribute to BBB function and integrity by regulating the expression of specific TJ proteins and other BBB transporters. Essentially, the TJ are the result of ostensible fusion between the outer lipid bilayers of neighbouring endothelial cells. Claudin, occludin, and junction adhesion molecules primarily form the composition of TJ, which serve to limit permeability between cells and to increase the barrier's electrical resistance. As a class of transmembrane proteins, two claudin extracellular loops undergo homophilic binding to loops from claudins on adjacent endothelial cells, forming the primary seal of the TJ. Distinct claudins isoforms regulate the diffusion of different sizes of molecules. To date, claudins 3, 5, and 12 are thought to be incorporated in the BBB [4, 5], while the presence of claudin-1 is still in debate [6]. For instance, claudin-5 knockout mice display abnormal endothelial cell TJ, increased BBB permeability to small molecules (<800 Da), and die shortly after birth [5]. Another transmembrane protein, occludin, is also implicated in the foundation of TJ. Similar to claudins, two occludin extracellular loops homophilically bind to occluding loops on a neighbouring cell, abetting in the formation of the TJ. In an occludin construct lacking the N-terminus and extracellular domains, an efficient permeability barrier failed to take shape with unblocked diffusion of several small markers and the presence of gaps, thus establishing the underlying significance of occludin proteins in the formation of TJ. Belonging to the immunoglobulin superfamily, junctional adhesion molecules (JAMs) with their single transmembrane domain are thought to contribute to the sealing capacity of TJ. However, the exact role of JAMs in the function of the BBB is still not fully understood. Adherins junctions (AJ) are typically found to be intermixed with TJ in the BBB. AJ are composed of the membrane protein cadherin whose extracellular domain homophilically binds cadherin on adjacent cells while the cytosolic domain is bound to catenins, which in turn are bound to the actin cytoskeleton of the cell, effectively joining neighbouring cells.

Unlike in the BBB where the barrier is localized at the level of the endothelial cells, the blood-cerebrospinal fluid (CSF) barrier is established by choroid plexus epithelial cells [7]. The choroid plexus is connected by apical TJ and consists of a capillary network, which is enclosed, in a single layer of epithelium cells [7, 8]. The choroid plexus epithelial cells limit paracellular diffusion and contain a secretory function producing the CSF. While the BBB may be the predominant site of transport for O_2_, glucose, and amino acids, the blood-CSF barrier plays a critical role in maintaining brain Ca^2+^ homeostasis [9]. The choroid plexus is also responsible for the entry of certain hormones into the CSF and also secretes insulin like growth factor-II (IGF-II) into the CSF [10]. The blood-CSF barrier also boasts of other active transport systems which aid in the efflux of certain solutes including iodide, thiocyanate and penicillin, and the neurotransmitter metabolites homovanillic acid and 5-hydroxyindoleacetic acid [11].

### 1.2. Functions of the BBB

The BBB limits the passage of large and hydrophilic solutes, while allowing small lipophilic molecules (O_2_, CO_2_, and hormones) to freely diffuse following concentration gradients. The BBB possesses specific transporters which are used to move complex nutrients such as glucose and amino acids into the brain. The BBB can also use receptor-mediated endocytosis to transport certain proteins such as insulin, leptin, and iron transferrin into the brain [12, 13].

#### 1.2.1. Regulation of Ion and Neurotransmitter Systems

The BBB plays a critical role not only in regulating the transport of macro- and micromolecules as mentioned above but also in the management of ion and neurotransmitter levels in the CNS and is the primary defence against neurotoxins. For instance, neuronal function and synaptic signalling relies on a stable environment containing optimal concentrations of specific ions such as potassium [K^+^]. In spite of a higher and fluctuating [K^+^] in the plasma akin to ~4.5 mM, the BBB helps maintain [K^+^] at ~2.4–2.9 mM in the CNS. Other major ions and systems regulated by the BBB include calcium (Ca^2+^), magnesium (Mg^2+^), and pH levels. The BBB also plays a major role in maintaining physiological levels of certain neurotransmitters, such as glutamate via excitatory amino acid transporters (EAATs), in the CNS. Additionally, the betaine/GABA transporter 1 (BGT1, SLC6A12), present in the brain microvessels, may play a role in the regulation of *γ*-aminobutyric acid (GABA) in the CNS [14, 15]. Notably, this compartmentalization of central and peripheral neurotransmitter pools by the BBB is important in the minimization of “cross talk” between these separate systems.

#### 1.2.2. Neurotoxins, Macromolecules, and Essential Nutrients

Taking into account that in the adult CNS steady neurodegeneration greatly overshadows neurogenesis [16], the sheltering of the CNS from endogenous and foreign toxins is of paramount importance. The TJ of the BBB provide an effective and stable barrier from potential toxins circulating in the peripheral blood, while a family of ATP-binding cassette (ABC) transporters in the BBB actively pump such toxins out of the brain. Total protein content in the CNS is inherently lower than plasma levels given the highly selective permeability of the BBB. Consequently, many plasma macromolecules such as albumin, prothrombin, and plasminogen, which can cause irreversible damage to nervous tissue resulting in apoptosis, are excluded. Furthermore, specific transporter systems such as the glucose transporter 1 (GLUT1) which is exclusive to the BBB and monocarboxylate transporter 1 (MCT1) facilitate the transport of glucose and monocarboxylates (i.e., lactate), respectively, as fuel for the brain [17]. The L1 and y^+^ systems, present ubiquitously in the BBB, provide transport for all essential amino acids into the CNS [18]. Five sodium dependent systems, ASC, A, LNAA, EAAT, and N, facilitate the efflux of nonessential AA (ASC, A), essential AA (LNAA), the excitatory acidic AA (EAAT), and nitrogen-rich AA (N) from the brain [4]. Larger neuroactive peptides and proteins including enkephalins [19], arginine-vasopressin (AVP) [20], and luteinizing-hormone releasing hormone (LHRH) can generally not pass the BBB and thus rely upon highly specific transporter systems to move from blood to brain and vice versa. Peptide transport system 1 (PTS-1) and PTS-2 mediate the efflux of enkephalins and AVP, respectively [21], from the brain while PTS-4 facilitates bidirectional transport of LHRH [4]. Other large proteins such as leptin [22], insulin and insulin-like growth factor [12], low-density lipoproteins (LDL) [23], and immunoglobulin-G (IgG) [24] also rely on receptor-mediated transport systems to cross the BBB.

In sum, the BBB serves two main functions: (i) establishment and maintenance of a specific and stable fluid environment to meet the rigorous needs of the CNS and (ii) protection of the CNS from potentially damaging material originating from both within and outside the confines of the body. The slightly imperfect nature of the BBB allows for the free diffusion of certain small essential water-soluble nutrients, while other complex nutrients rely on highly selective transport systems to enter the brain. Therefore, considering the central role of the BBB in protecting the CNS against neurotoxic compounds, there has been growing interest in the understanding of the BBB function in neuropsychiatric disorders.

### 1.3. Neurovascular Unit

The neurovascular unit (NVU) was initially defined as “interactions between circulating blood elements and the blood vessel wall, extracellular matrix, glia, and neurons” [25] but has recently developed to incorporate other structures including pericytes and microglia (detailed anatomy and organization is reviewed in [26, 27]). Due to the amalgamation of these structures, the NVU is considered the site of the coupling of neuronal activity and cerebral blood flow [28, 29]. The various components of the NVU are intricately linked to one another, and this relationship is facilitated by adhesion molecules (including cadherins and integrins) and gap junctions [30–32], which in conjunction with ion channels aid in the movement of various ions such as calcium and potassium, and also other neuromodulators (ATP) [33, 34]. The interlink between neuronal and vascular components is genetically tied as during early embryogenesis neural progenitor cells (originating from neural tube) and vascular progenitor cells (originating from neural plate) are positioned in close proximity [35–37]. Due to their position relative to one another, both neural and vascular cells are exposed to similar factors and both respond to vascular endothelial growth factor (VEGF) and nerve growth factor (NGF) [38, 39]. The various components of the NVU all play a distinct and specific role in maintaining the functionality of the NVU [30, 40]; however the exact role of each component is still yet to be elucidated [37, 41].

Exploring the relationship between neurological conditions and NVU dysfunction is still in its infancy; however indirect and epidemiological data does suggest a role for NVU dysfunction in psychiatric conditions such as major depressive disorder (MDD). A study examining endothelial dysfunction via the relative uptake ratio (RUR) of blood flow in the brachial artery following hyperemic challenge found a significantly lower RUR in patients with MDD or minor depressive disorder as compared to healthy controls, implying impaired vascular endothelial function [42]. Another study exploring apoptotic activity in the endothelium (% of apoptotic nuclei in human umbilical vein endothelial cells) found a significantly greater amount of apoptotic nuclei in patients with MDD when compared to healthy controls [43]. Epidemiological studies also point towards a role for vascular endothelial impairment in MDD. A meta-analysis encompassing 16,221 studies found an increased risk for MDD in those with major vascular diseases including diabetes, cardiovascular disease, and stroke [44].

## 2. Models of BBB Disruption in Neuropsychiatric Disorders 

### 2.1. BBB in Alzheimer's Disease and Multiple Sclerosis

#### 2.1.1. Alzheimer's Disease

Alzheimer's disease (AD) is characterized by a progressive decline in cognitive function with an onset of >65 years old in most cases. Biologically, AD has been associated with defects in the neurovascular system, accumulation of amyloid-*β* (A*β*; neurotoxin) on and around blood vessels as well as in the brain parenchyma, and the presence of neurofibrillar tangles (NT) [4, 45, 46] and hyperphosphorylated tau [47]. The role of A*β* in AD is the most studied and well understood. Notably, it has been recently shown that peripheral circulating A*β* is transported into the brain via the receptor for advanced glycation end products (RAGE) [48]. Normally expressed in relatively low levels, the expression of RAGE in the BBB is greatly amplified in response to the accumulation of RAGE ligands including A*β* [48, 49]. This A*β*/RAGE interaction in the BBB may lead to increased transportation of circulating A*β* into the CNS, resulting in a NF-*κ*B mediated activation of endothelial cells and the release of proinflammatory cytokines. It has been demonstrated that binding of A*β*/RAGE at the luminal membrane of the BBB can destroy RAGE expressing neurons through oxidative damage. The clearance of A*β* from the brain is facilitated by lipoprotein receptor-related protein 1 (LRP1). Numerous studies using both animal models and human patients with AD show that A*β* clearance is impaired in these cases [49–53]. For instance, LRP1 functions to transport A*β* into the periphery vascular system whereupon soluble LRP1 (sLRP1) facilitates the total systematic clearance of A*β* from the body via the kidney and liver. The role of LRP2 is not well understood but is hypothesized to utilize apolipoprotein J (APOJ) to facilitate the transfer of A*β* out of the brain [54]. Moreover, the ATP-binding cassette (ABC) family of transporters have also been implicated in A*β* clearance. ABCB1 (P-glycoprotein, P-gp), the product of the MDR1 gene, is the best known and best studied of these transporters. Most commonly found in the BBB, several* in vitro *and* in vivo *studies have found ABCB1 to clear A*β* from the albuminal to the luminal side of the membrane [55]. In MDR1 transfected pig kidney epithelial cells, the transport of A*β*_40_ and A*β*_42_ was significantly decreased in the presence of cyclosporine A (ABCB1 inhibitor) [56]. Additionally, following the injection of labelled A*β*_40_ and A*β*_42_ into ABCB1 knockout mice, the clearance rate of A*β* was found to be half that of the wild type [57]. In addition to faulty BBB clearing mechanisms in the pathology of AD, recent evidence using APP23 transgenic mice overexpressing mutant human APP, the precursor of A*β*, suggests that the BBB may be susceptible to peripherally induced inflammation [46]. For instance, Takeda et al. administered a peripheral LPS injection to APP transgenic mice and observed a greater increase in inflammatory levels in the brain interstitial fluid, which was accompanied by abnormalities in food intake, social behaviour, and basal activity [46]. In summary, various models of AD suggest that BBB dysfunction is associated with abnormal A*β* clearance and increased permeability and subsequent influx of proinflammatory substances into the CNS.

#### 2.1.2. Multiple Sclerosis

Multiple sclerosis (MS), a brain disorder characterized by extensive damage to the myelin sheath, presents a wide host of symptoms including but not limited to numbness and weakness in limbs, visual impairments, electric shock sensations, tingling and/or pain across the body, and cognitive impairment. While the exact cause of MS remains unknown, there is still a debate whether or not MS is an autoimmune disease, as classically held, or if it is in reality a neurodegenerative disorder [58, 59]. With respect to the autoimmune aspect of MS, the BBB is responsible for the regulation of immune cell transport and inflammatory pathway mediator activity from the periphery into the CNS. Under physiological conditions, few leukocytes are present in the CNS but in response to injury and/or disease peripheral leukocytes are thought to enter the cerebral spinal fluid (CSF), the parenchymal perivascular space, and the subarachnoid space [2, 18, 19]. In the experimental autoimmune encephalomyelitis (EAE) model of MS, it has been shown that aggressive CD4+ T lymphocytes accumulate in the brain via the BBB and blood-CSF barrier [60–62]. A subset of these T lymphocytes have been reported to exert immunosurveillance in the CNS while another subset is implicated in the destruction of neurons. The regulation and transport of immune cells and other mediators across the BBB and blood-CSF barrier are thus thought to be implicated in the pathophysiology of MS. An imaging study using dynamic contrast-enhanced MRI (DCE-MRI) noted an increase in BBB permeability, as measured by K^trans⁡^, in the periventricular normal appearing white matter (NAWM) in patients with MS [63]. Notably, immunomodulatory treatment (with *β*-interferon or glatiramer acetate) aided in the gradual decrease of BBB permeability following a relapse episode. Considering that *β*-interferon has been shown to stabilize the barrier on brain capillary endothelial cells* in vitro *[64], this study provided strong evidence that abnormalities in the BBB function may be associated with the neurobiology of MS. Notably, a recent* in vitro* study that exposed human brain microvascular endothelial cells (BMECs) to serum from patients with relapse-remitting MS (RRMS) found that serum from patients with RRMS lowered claudin-5, an integral TJ protein expression, and decreased transendothelial electrical resistance [65]. Together, these clinical and preclinical studies indicate that an increase in BBB permeability may occur soon after the flare-ups observed in MS. In addition, preliminary yet encouraging data suggest that successful anti-inflammatory treatment may speed up the rate of closing of the BBB.

#### 2.1.3. The Role of Matrix Metalloproteinase-9 on BBB Function

Matrix metalloproteinases (MMPs) encompass a large family of proteases which are typically produced in a latent form and upon activation by inflammatory stimuli regulate pathophysiological pathways including the regulation of growth factors, death receptors, and various other signalling molecules [66, 67]. The effects of MMPs are diverse and depend on a host of factors such as location, time, and surrounding environment and thus some MMPs can engage in opposite functions at different points in time. For instance, MMPs have been implicated in angiogenesis, neurogenesis, axon growth, tissue repair, myelinogenesis, and apoptotic protection [68–70]. Notably, the promoter region of MMP9 includes a binding region for activator protein-1 (AP1) and NF-*κ*B, both of which are involved in key inflammatory pathways and thus linking neuroinflammation and MMP9 [67]. Upon the induction of the neuroinflammatory pathway, MMP9 along with MMP2 and MMP3 can facilitate the proteolysis of the basal lamina, TJ, and extracellular matrix resulting in increased BBB permeability [71, 72]. Inhibitors of MMPs have been shown to restore BBB integrity [73]. In individuals experiencing an exacerbation of MS, MMP9 was found to be elevated in the CSF [74] and treatment with prednisolone was found to restore BBB integrity resulting in a decrease of MMP9 levels in the CSF [75]. Furthermore, in an EAE model of MS in which demyelination is associated with neuroinflammation, treatment with the MMP inhibitor GM-6001 halted the progression of EAE in mice [76].

Accumulation of A*β* endogenously induces the secretion of MMPs in microglia and astrocytes as a part of the neuroinflammatory pathway [67, 77]. Plasma MMP9 levels are elevated in patients with AD [78]. PCR and immunohistochemistry data show accumulation of a latent/inactive form of MMP9 in the hippocampus of patients with AD [79], which is postulated to be associated with less degradation of A*β* plaques in the brain. In addition, A*β*-induced cognitive impairment and neurotoxicity were significantly alleviated in MMP9 homozygous K/O mice and with administration of MMP inhibitors [80]. Together, these studies indicate an important role of MMP9 in AD and MS via BBB dysfunction.

### 2.2. BBB in Schizophrenia

The role of BBB dysfunction in psychiatric conditions has been far less studied. Some studies have investigated “blood-CSF barrier dysfunction” as measured by CSF-to-serum albumin ratio. Evidence of increased CSF-to-serum albumin ratio has been reported in individuals with schizophrenia (SCZ) [81–83], bipolar disorder (BD) [84], and a mixed sample of inpatients with mood and SCZ spectrum disorders [85]. Given that albumin is not synthesized in the CSF, all albumins present in the CSF originated from the peripheral blood compartment. Thus, these findings of elevated CSF-to-serum albumin in mood and SCZ subjects have been interpreted as potential blood-CSF or BBB dysfunction. A recent controversial study [86] proposed a link between BBB dysfunction and SCZ based on two indirect findings: (a) worse scores in the Cambridge Neurological Inventory in SCZ subjects who were positive for anti-NMDA receptor autoantibodies and had past history of birth complications or head trauma (used as proxies of BBB disruption) and (b) behavioural changes in ApoE −/− mice (known to display BBB deficiency) after injection of Ig fractions from NMDAR-autoantibodies (NMDAR-AB) seropositive (IgM, IgG, and IgA) subjects compared to serum from control subjects. However, this study has been criticized [87] by (i) using retrospective data to determine birth complications and history of head trauma and assuming that these retrospective events disturbed BBB integrity; (ii) by providing no confocal microscopy images pertaining to their NMDA receptor immunostaining in the presence of NMDAR-AB, thus calling into question their immunostaining results by pointing to other studies [88, 89] which utilized anti-NMDR encephalitis antibodies to visualize NMDAR internalization with confocal microscopy and could not draw the same conclusions; and (iii) by suggesting that the study needed to prove that the injection of patients' IgG reached the brain, bound to NMDAR, and altered receptor levels and functions before drawing strong conclusions using the ApoE −/− mice data.

Therefore, while the study of BBB in psychiatric disorders is still in its infancy, there is converging data showing that SCZ and BD are associated with increased CSF-to-serum albumin ratio.

## 3. Why BBB Disruption May Be Associated with Bipolar Disorder? 

Like most major neuropsychiatric disorders, BD has also been heavily linked with inflammatory processes. In fact, increased neuroinflammation is thought to mediate, at least in part, the cognitive decline as well as the abnormalities observed in gray and white matter content in individuals with BD. In addition, several cohort studies have now demonstrated that BD is associated with excessive mortality rates [90–92]. Compared to the general population, individuals with BD die on average 9 years younger [93], but, more importantly, these striking elevated mortality rates are primarily due to death from natural causes including cardiovascular, respiratory, diabetes, and infectious diseases, all of which have been associated with increased inflammation [93–95]. Below, we propose a novel model where disruption in the BBB is associated with less protection and subsequently more influx of inflammatory material from the periphery to the brain of individuals with BD.

### 3.1. Inflammation and Oxidative Stress in Bipolar Disorder

Several lines of evidence indicate that BD is associated with increased inflammation and oxidative stress. For instance, the monocyte-T cell theory of mood disorders implicates the inflammatory response system (IRS) as a primary contributor to the neurobiology of BD [96]. This theory is supported in part by evidence of increased levels of proinflammatory cytokines including IL-1, IL-6, and TNF-*α* in plasma [97, 98], abnormal expression of proinflammatory genes in circulating monocytes [99], and evidence that psychotropics can modulate the immune system [98–101]. Activation of the immune system is linked with neuroinflammation through activation of microglia which is a central player in neuroinflammatory pathways [97]. A recent PET imaging study using [^11^C]-(*R*)-PK11195 found greater [^11^C]-(*R*)-PK11195 binding potential in the right hippocampus and a similar nonsignificant trend in the left hippocampus of bipolar type I subjects, suggesting increased microglial activity and neuroinflammation in these brain areas. Notably, oxidative damage to RNA [102] and decreased expression of growth associated proteins [103], both believed to be involved in neuroinflammation [104], have been observed in postmortem hippocampal samples from BD subjects. Disruption of mitochondria, responsible for the regulation of apoptosis and intracellular calcium levels, has been increasingly implicated as a contributing factor in the oxidative stress facet of BD perhaps through decreased activity of mitochondrial complex I [105]. Moreover, studies conducted in the peripheral blood have consistently found increased markers of oxidative damage to lipids, RNA, and DNA in BD [106, 107].

### 3.2. Oligodendrocyte and Myelin Damage in Bipolar Disorder

Oligodendrocytes facilitate the formation and stability of neural circuits by insulating axons with myelin sheath. In the last several years, there has been increasing attention to changes in white matter and oligodendrocyte structure/function in BD. For instance, oligodendrocyte-specific mRNA markers including OLIG2, SOX10, GALC, MAG, PLP1, CLDN11, MOG, ERBB3, and TF were found to be downregulated in the brain of individuals with BD [108]. Uranova et al. used electron microscopy to analyze ultrastructural altercations in oligodendrocytes in the prefrontal cortex of individuals with BD [109]. The oligodendrocyte cells in BD were found to be surrounded by astroglial cells and displayed strong signs of apoptosis and necrosis. In this qualitative study, apoptosis was characterized by nuclear chromatin aggregation, cell shrinkage, and the preservation of organelles while necrosis was characterized by chromatin condensation, cell swelling, and membrane lysis of organelles. Previously, this group described a decrease in oligodendrocyte density in layer VI of BD patients (31%) [110], further implicating oligodendrocyte disruption in the pathophysiology of BD. Furthermore, several imaging, genetic, and postmortem tissue analyses have shown myelin abnormalities in BD subjects [108, 111–114], establishing a link between oligodendrocyte dysfunction and myelin damage in BD.

### 3.3. Implication of Inflammation and Oxidative Stress in the Treatment of Bipolar Disorder

One of the key questions in BD research has been the extent to which available treatments may reverse/prevent inflammation and oxidative stress. While an extensive review of the effects of pharmacological and nonpharmacological treatments on inflammation and oxidative stress is beyond the objective of the present paper, there is growing evidence that mood stabilizing and antidepressant agents possess anti-inflammatory and antioxidant properties (as reviewed in [115, 116]). Lithium, the hallmark treatment of BD, was shown to aid in the defence against oxidative stress by upregulating mitochondrial complexes I and II [117]. Relevant to the notion that lithium can protect against ROS-induced damage, previous studies have shown that oxidative stress can effect BBB permeability, particularly by affecting the integral TJ protein occludin [118, 119]. Administration of tempol, a ROS scavenger, to *λ*-carrageenan-induced peripheral inflammatory pain (CIP) rats attenuated (14)C-sucrose and (3)H-codeine uptake in the brain and provided protection to occludin, thus preserving BBB integrity [120]. Future studies investigating the ability of lithium to protect against BBB disruption are warranted.

Lithium also downregulates the arachidonic acid-prostaglandins (PGs) pathway [121, 122] which has been implicated with neuroinflammation [123, 124]. More specifically, chronic lithium treatment resulted in decreased AA to PGs turnover, decreased activity of cyclooxygenase-2 (COX-2), the enzyme responsible for converting AA to PGs, and PG-E_2_ concentration in rat brain [125]. Another preclinical study showed that lithium treatment significantly increased levels of 17-hydroxy-docosahexaenoic acid [126], which possesses known anti-inflammatory properties [127, 128]. Furthermore, several* in vitro *an* in vivo *studies have shown that lithium treatment results in the attenuation of proinflammatory cytokines including TNF-*α* [129–131], IL-1*β* [132–134], IL-6 [135–137], and interferon-*γ* (INF-*γ*) [138–140] while increasing the secretion of the anti-inflammatory cytokines IL-2 [141–143] and IL-10 [134, 140, 144]. With respect to oligodendrocyte function, lithium treatment has been shown to increase oligodendrocyte proliferation and increase myelination of optic nerves in mice [145].

In summary, there is overwhelming data pointing towards inflammatory and oxidative stress modulation by lithium and other psychotropic agents. Given that inflammation and oxidative stress have been associated with disruption in the BBB integrity, a natural next step for future studies is to test whether lithium and/or other mood stabilizing agents used in the treatment of BD may protect against BBB damage.

### 3.4. A Novel Model of BBB Disruption in Bipolar Disorder

**Figure 1:**
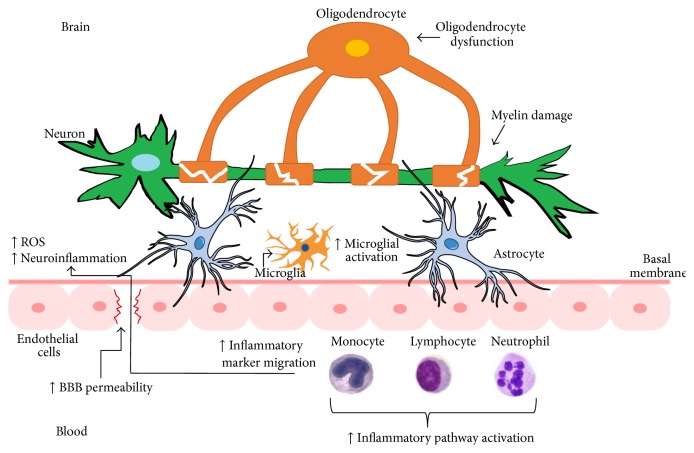
Proposed model of blood-brain barrier (BBB) disruption in bipolar disorder. Increased BBB permeability through the endothelial cells (pink) and basal membrane (dark pink) may facilitate increased migration of inflammatory molecules into the brain. Activation of microglial cells (light orange) and an increase in reactive oxygen species (ROS) would amplify neuroinflammatory processes and ultimately induce damage in the myelin sheath, either directly via lipid/protein oxidation or indirectly via oligodendrocyte dysfunction (dark orange).

Decades of research has implicated increased peripheral inflammation and oxidative stress, as well as oligodendrocyte and white matter changes in the pathophysiology of BD. This is in line with a number of cohort studies showing increased mortality rates due to general medical conditions associated with inflammation and oxidative stress. Further evidence is provided by studies showing that first-line treatments for BD, such as lithium, can modulate inflammatory and oxidative stress pathways. More recently, imaging and postmortem studies have provided evidence of increased neuroinflammation in BD through excessive microglial activation. Considering the close anatomical proximity of microglia, oligodendrocytes, and astrocytes to the BBB, and the increasing attention of BBB disruption in other neuropsychiatric conditions, such as AD, MS, and SCZ, we propose a novel model of BBB dysfunction in BD wherein transient or persistent loss of BBB integrity is associated with decreased CNS protection and increased permeability of proinflammatory (e.g., cytokines, reactive oxygen species) substances from the peripheral blood into the brain. This will trigger the activation of microglial cells and promote localized damage to oligodendrocytes and the myelin sheath, thereby compromising myelination and neural circuit integrity (Figure 1).

While we could not identify a study that directly examined the BBB integrity in BD, a recent study found increased levels of MMP9, which increases BBB permeability during proinflammatory states (see Section 2.1.3), in bipolar depression [146]. In addition, both manic and depressive episodes are associated with increased levels of proinflammatory cytokines [147] and, therefore, it is conceivable that BD subjects may experience a transient increase in the BBB permeability during each major mood episode. Also, it is well established that most drugs of abuse disrupt the BBB integrity [148, 149]. Given the exceeding rates of drug abuse in individuals with BD, it is also likely that excessive drug use can contribute to the disruption in BBB permeability in a substantial proportion of individuals with BD. This is in line with an elegant twin study showing that peripheral proinflammatory states in BD are primarily the result of environmental as opposed to genetic factors [150].

### 3.5. Future Directions

It is imperative to test this model by further analyzing the role of the BBB in BD. Currently, at least a couple of brain imaging techniques are available to test the hypothesis of disrupted BBB structure or function directly in individuals with BD. One possibility would be the use of dynamic contrast-enhanced MRI (DCE-MRI) as a method for studying BBB disruption* in vivo* [151]. Another available technique is the use of [^11^C]-verapamil to study the function of the P-glycoprotein (Pgp) transporter at the blood-brain barrier (BBB) with PET [152]. Finally, the use of* in vivo* and* in vitro* preclinical models may be particularly useful to test whether lithium and other medications commonly used in the treatment of BD can reverse and/or prevent BBB damage. If a link between BD and BBB disruption is established, this would not only advance the knowledge on the neurobiology of BD but also open numerous possibilities to investigate new treatment pathways (e.g., MMP inhibitors [153], ROS scavengers [120]) for this devastating major mental illness.
